# Elevated miR-16-5p induces somatostatin receptor 2 expression in neuroendocrine tumor cells

**DOI:** 10.1371/journal.pone.0240107

**Published:** 2020-10-12

**Authors:** HanHee Jo, Yusun Park, Jisu Kim, Hyeonjeong Kwon, Taehun Kim, JongSook Lee, Jae-Chul Pyun, Misu Lee, Mijin Yun

**Affiliations:** 1 Division of Life Sciences, College of Life Science and Bioengineering, Incheon National University, Incheon, South Korea; 2 Department of Nuclear Medicine, Severance Hospital, Yonsei University College of Medicine, Seoul, South Korea; 3 Department of Materials Science and Engineering, Yonsei University, Seoul, South Korea; University of Hawai'i at Manoa, UNITED STATES

## Abstract

Somatostatin analogs, which are used to treat neuroendocrine tumors, inhibit hormone secretion or promote tumor shrinkage; however, their efficacy varies between patients, possibly because of differential expression of somatostatin receptors (SSTRs) in tumors. In this study, we evaluated the regulatory mechanism underlying the expression of SSTR2, the main octreotide target. Thirty miRNAs were found to be dysregulated in neuroendocrine cells (INS-1 cells) incubated with octreotide compared to that in placebo-treated cells. Among the upregulated miRNAs, miR-16-5p was elevated after short-term octreotide treatment. We conducted *in vitro* experiments to determine whether the expression of miR-16-5p was associated with the regulation of SSTR2 expression and affected octreotide sensitivity in INS-1 cells. Overexpression of miR-16-5p by transfected mimics induced upregulation of SSTR2 expression. Additionally, the expression of miR-16-5p further enhanced octreotide-induced reduction in cell proliferation in both two- and three-dimensional culture of INS-1 cells. Thus, our results reveal the mechanism underlying SSTR2 expression regulation and may aid in developing therapeutic approaches for enhancing the response to octreotide, particularly in patients unresponsive to SSTR2-targeted somatostatin analog treatment.

## Introduction

Somatostatin (somatotropin release-inhibiting hormone, SST), a small polypeptide hormone produced in the hypothalamus [1], regulates endocrine function mainly by suppressing the release of hormones and neurotransmitters and inhibits tumor growth by regulating tumor cell survival and angiogenesis [2]. SST binds with high affinity to somatostatin receptors (SSTR1–5), which are specific G protein-coupled receptors present on cell surfaces [3]. Neuroendocrine tumors (NETs) are a heterogeneous group of neoplasms with a low growth rate in most cases; however, they occasionally secrete hormones that cause specific clinical syndromes, resulting in significant disability and decreased quality of life [4]. SSTR mRNA and protein expression patterns in NETs have been studied extensively, and SSTRs have been used as potential diagnostic and therapeutic targets because of their widespread expression and function in NETs.

SST analogs (SSAs) have been developed and widely investigated [3]; for instance, octreotide and lanreotide are suitable for clinical applications because of their considerably longer half-life compared with that of natural SST. Similar to natural SST, SSAs bind to SSTRs; however, SSAs show different binding affinities for various receptors. Octreotide and lanreotide bind to SSTR2 with high affinity, followed by SSTR5, and activate inhibitory signals [5, 6]. Thus, a positive correlation between the anticancer efficacy of octreotide and SSTR2 expression exists in NETs [2]. However, few studies have investigated mechanisms for enhancing SSTR expression and thereby increasing the anticancer effect of SSAs. Although acute administration of SST induces the activation of various inhibitory signals, the initial response against SSA is diminished. Continuous exposure to SSA reduces the initial drug effects via processes such as degradation, internalization, and phosphorylation of SSTRs [7].

MicroRNAs (miRNAs) are a class of non-coding RNAs comprising 21–25 nucleotides. miRNAs have been reported to be closely associated with the regulation of different signaling pathways and several biological functions, including cell proliferation, differentiation, survival, and metabolism. miRNAs function by binding to complementary target mRNAs, resulting in mRNA translational inhibition or degradation [8]. Thus, several miRNA-targeting therapeutics have been developed and are being tested via clinical trials. Recently, miRNAs differentially expressed between SSA responders or non-responders in growth hormone-secreting pituitary adenomas have been identified [9]. However, the effects of miRNA-mediated control of SSTR2 expression and the subsequent effect on SST drug sensitivity in NETs remain poorly understood.

In this study, we identified novel miRNAs involved in the early response to octreotide treatment and investigated SSTR2 expression during this early response. This study provides insight into the mechanism of octreotide, which may be useful for enhancing its anticancer effects, particularly in octreotide non-responder patients with NETs.

## Materials and methods

### Cell culture

The INS-1 rat insulinoma cell line and GH3 rat pituitary GH- and PRL-producing cell line were purchased from Addexbio (San Diego, CA, USA) and the Korean Cell Line Bank. Both cell lines expressed SSTR2 (S1 Fig). INS-1 cells and GH3 cells were cultured in RPMI-1640 (Gibco, Grand Island, NY, USA) and Dulbecco’s Modified Eagle Medium supplemented with 10% fetal bovine serum, 0.05 mmol/L 2-mercaptoethanol, 100 U/mL penicillin, and 100 μg/mL streptomycin and maintained at 5% CO_2_ and 37°C. HeLa cells were purchased from the Korean Cell Line Bank and grown in Dulbecco’s Modified Eagle Medium supplemented with 10% (v/v) fetal bovine serum and 1% (v/v) penicillin–streptomycin. Octreotide was purchased from TOCRIS Bioscience (Bristol, UK) and dissolved in deionized water to prepare a 1 mM stock solution. For 3-dimensioanl culture, Costar® Ultra-Low attachment multi-well plates were purchased from Sigma-Aldrich Korea (St. Louis, MO, USA) and used as described previously [10].

### RNA extraction and miRNA synthesis

Total RNA was extracted using TRIzol reagent (Life Technologies, Carlsbad, CA, USA) according to the manufacturer’s instructions. RNA concentrations were measured using a NanoDrop spectrophotometer (DeNovix, Wilmington, DE, USA). miRNAs were synthesized from 500 ng of total RNA using an miScript II RT Kit (Qiagen, Hilden, Germany) according to the manufacturer’s instructions.

### Library preparation, sequencing, and data analysis

For control and test RNAs, a library was constructed using the NEBNext Multiplex Small RNA Library Prep Kit (New England Biolabs, Ipswich, MA, USA) according to the manufacturer’s instructions. Briefly, 1 μg of total RNA from each sample was used to ligate the adaptors, after which cDNA was synthesized using reverse-transcriptase with adaptor-specific primers. PCR was performed for library amplification, and then the libraries were purified using a QIAquick PCR Purification Kit (Qiagen) and AMPure XP beads (Beckman Coulter, Brea, CA, USA). The yield and size distribution of the small RNA libraries were assessed by high-sensitivity DNA analysis on an Agilent 2100 Bioanalyzer (Agilent Technologies, Santa Clara, CA, USA). High-throughput sequences were produced by single-end 75 sequencing using the NextSeq 500 system (Illumina, San Diego, CA, USA). Sequence reads were mapped using the Bowtie 2 software tool to obtain the BAM file (alignment file). A mature miRNA sequence was used as a reference for mapping. Read counts mapped onto the mature miRNA sequence were extracted from the alignment file using bedtools (v2.25.0) and Bioconductor which uses R (version 3.2.2) statistical programming language (R development Core Team, 2011). The read counts were then used to determine the expression levels of miRNAs. The quantile normalization method was used to compare samples. miRWalk 2.0 was used for miRNA target analysis. Functional gene classification was performed using DAVID (http://david.abcc.ncifcrf.gov/).

### Transfection and treatment

HeLa cells (1 × 10^5^cells/well) were plated in 6-well plates. After 24 h, 1 μg hSSTR2 or control plasmid (Sino Biological, Inc., Eschborn, Germany) was transfected using RNAiMAX transfection reagent (Invitrogen, Carlsbad, CA, USA) according to the manufacturer’s instructions. After an additional 24 h of incubation, an miRNA inhibitor was used to treat the HeLa cells. INS1 cells (1 × 10^5^ cells/well) were cultured in 6-well plates. A commercial miR-16-5p mimic (synthesized by Genolution, Seoul, South Korea) with the following sequence, sense, 5′-UAGCAGCACGUAAAUAUUGGCG-3′ and antisense, 5′-CGCCAAUAUUUACGUGCUGCUA-3′ or negative control siRNA (Genolution) was transfected into INS1 cells. Transfection was performed using RNAiMAX transfection reagent (Invitrogen) according to the manufacturer’s instructions. After 24–48 h incubation, further analyses were performed.

### Real-time quantitative PCR (RT-qPCR)

To analysis miRNA expression, RT-qPCR was conducted on a C1000™ Thermal Cycler (Bio-Rad Laboratories, Hercules, CA, USA) using an miScript SYBR Green PCR Kit with miScript miRNA PCR Arrays (Qiagen). Gene expression levels were normalized to miRTC expression levels for the corresponding miRNA samples. The following target miRNAs were quantified by miScript Primer assays (Qiagen), rno-miR-16-5p (cat no. MS00033229; mature miRNA with the sequence UAGCAGCACGUAAAUAUUGGCG), and miRTC (MS00000001). cDNA was synthesized from 500 ng of total RNA using ReverTra Ace qPCR RT Master Mix with gDNA Remover (Toyobo, Osaka, Japan). Quantitative RT-PCR was performed using SYBG Green Real-time PCR Master Mix (Toyobo). Gene expression levels were normalized with beta-2 microglobulin (B2M) mRNA expression levels of corresponding cDNA samples. All PCR primers were purchased from Bioneer (Daejeon, Korea). The following primers were used: r*ARRB1* (Forward 5′-ACCTGGATGTCTTGGGTCTG-3′ -Reverse 3’-TAGCCGAGTCAGTGGCTTCT-5′, h*SSTR2* (forward 5′-TGAGAAGAAGGTCACCCGAAT-3′, reverse 3′-AGGACCACCACAAAGTCAAAC-′) and h*B2M* (forward ′-TTACTCACGTCATCCAGCAGA-3′, reverse 3’-AGAAAGACCAGTCCTTGCTGA-′).

### Cell viability assay and flow cytometry analysis

Cell viability was assessed using the CCK8 assay. INS1 cells were seeded at 1 × 10^5^ cells/ml in a 96-well plate, incubated for 24 h, and treated under various conditions. To measure cell viability, 10 μL/well of CCK8 reagent (Dojindo Laboratories, Kumamoto, Japan) was added to each well and the plates were incubated in a humidified incubator at 37°C for 30 min. Absorbance at 440 and 640 nm was measured using a Spectra Max190 microplate reader (Molecular Devices, Sunnyvale, CA, USA). Cell viability for 3D spheroids was assessed in a CellTiter-Glo® 3D Cell Viability Assay (Promega, Madison, WI, USA). To quantify apoptosis, double staining was performed following the protocol as described in Annexin V-FITC Apoptosis Detection Kit (BD Pharmingen™, Franklin Lakes, NJ, USA). INS1 cells were collected after incubation with mimic miR-15-5p and octretodie and analyzed as described previously [11].

### Immunocytochemistry

Immunocytochemistry was performed as previously described [10]. Cells fixed in 4% paraformaldehyde were blocked with 3% bovine serum albumin for 30 min at room temperature (25–27°C) and incubated with SSTR2 primary antibody (clone UMBI; 1:500; Abcam, Cambridge, UK) at 4°C overnight. The cells were washed, incubated with the appropriate fluorescence-conjugated secondary antibody (1:200; Invitrogen) for 1 h at room temperature, and counterstained with Hoechst (Invitrogen). The coverslips were then mounted, and the cells were observed under a light microscope. Images were recorded using an Olympus BX53 microscope with Olympus Cell Sens software (Tokyo, Japan).

### Western blotting

Western blotting was performed as previously described [10]. The following primary antibodies were used: SSTR2 (clone UMBI; 1:500; Abcam) and β-actin (C4; 1:5000; Santa Cruz Biotechnology, Dallas, TX, USA). As secondary antibodies, horseradish peroxidase-labeled rabbit IgG and mouse IgG antibodies were used (GeneTex, Irvine, CA, USA). Protein levels were semi-quantified via densitometry by analyzing the blots using Image Lab software (Bio-Rad Laboratories).

### Statistical analysis

A paired two-tailed Student's *t*-test was used to detect significant differences between two sets of data. The differences were considered as statistically significant when p values were <0.05.

## Results

### Identification of differentially expressed miRNAs in INS-1 cells during the early response to octreotide treatment

**Fig 1:**
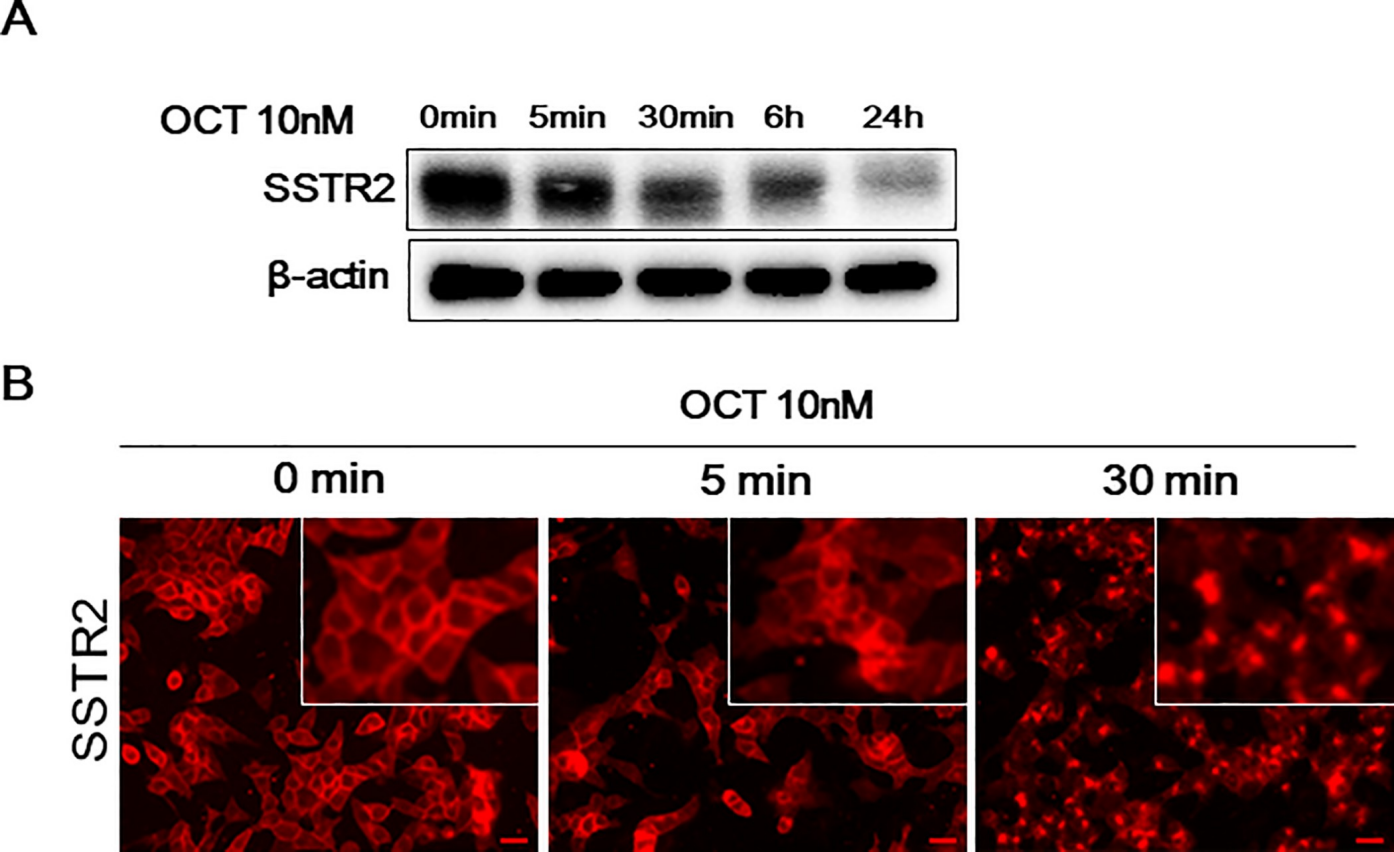
Expression of SSTR2 after octreotide treatment in INS-1 cells. (A) INS-1 cells were incubated with 1 μmol/L octreotide. After the indicated time, SSTR2 and β-actin levels were analyzed by western blotting. The images shown here are cropped and the full-length original blots are shown in S6 Fig. (B) INS-1 cells were plated on coverslips in 24-well plates. On the next day, the cells were incubated with 1 μmol/L octreotide for the indicated time. Cells were fixed and processed for immunofluorescence staining of SSTR2 protein. Scale bar: 20 μm.

**Fig 2:**
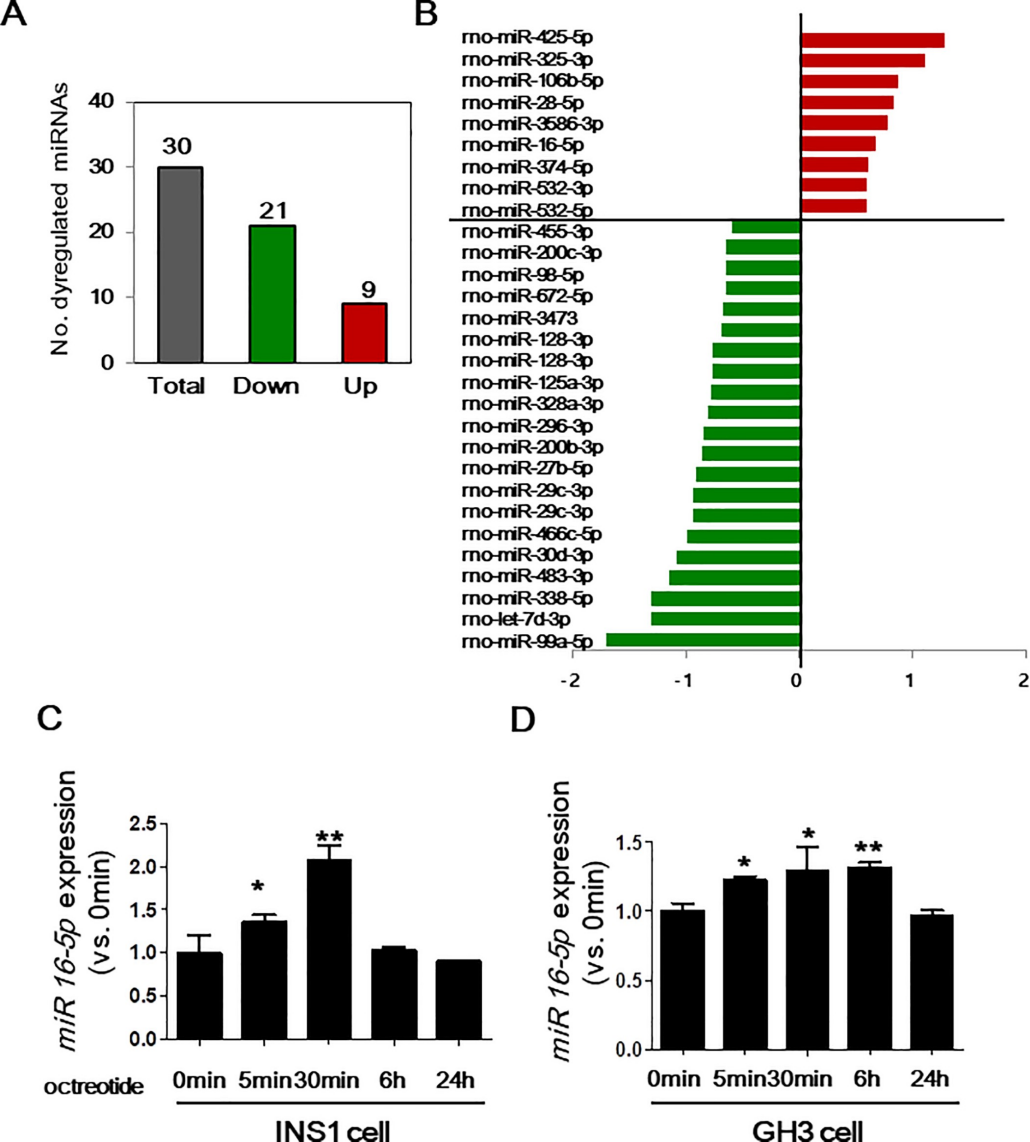
Microarray analysis of miRNA expression. miRNA profiling was performed to determine the (A) number and (B) profile of differentially expressed miRNAs. Red and green indicate significantly upregulated or downregulated miRNAs, respectively. INS-1 cells (C) and GH3 cells (D) were incubated with 1 μmol/L octreotide. After the indicated incubation time, total RNA was isolated, after which miR-16-5p levels were measured by qRT-PCR and normalized for input RNA using control miRNA (U87) with the 2^−ΔΔCt^ method. Data represent the mean of three independent experiments ± SD; *p < 0.05, **p < 0.01.

**Fig 3:**
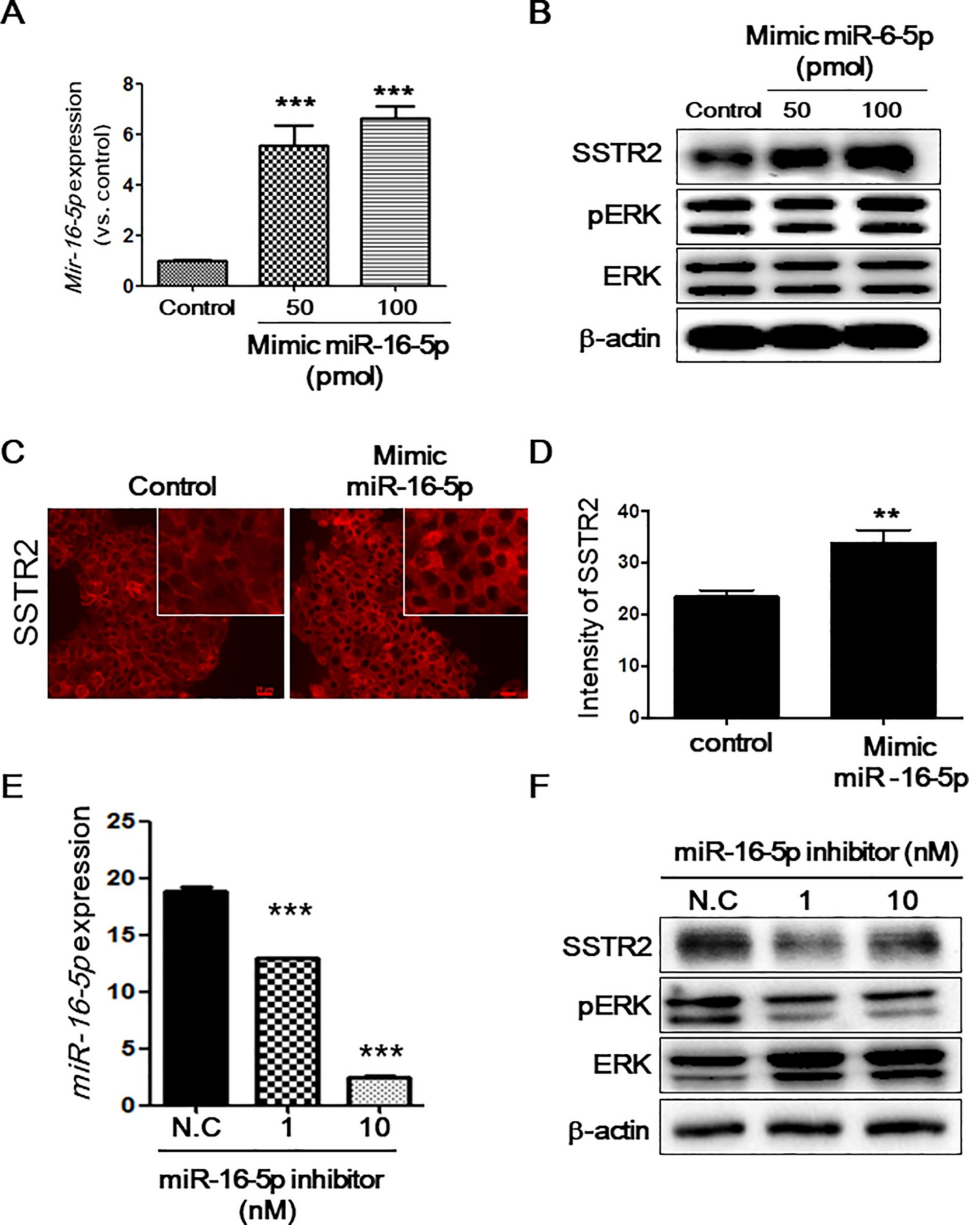
Expression of SSTR2 after treatment with miR-16-5p mimic or inhibitor in INS-1 cells. INS-1 cells were transfected with 50 or 100 pmol of mimic miR-16-5p or control miRNA. (A) miR-16-5p levels were examined by RT-qPCR at 48 h after transfection. miRNA expression of target genes was normalized for input RNA using endogenous control miRNA (U87) and calculated by the 2^−ΔΔCt^ method. (B) SSTR2, phospho-ERK (Thr202/Tyr204), ERK, and β-actin levels were analyzed by western blotting. The images shown here are cropped and the full-length original blots are shown in S7 Fig. (C) INS-1 cells were plated on coverslips in 24-well plates and transfected with 100 pmol of mimic miR-16-5p or control miRNA. After 48 h, the cells were fixed and processed for immunofluorescent staining of SSTR2 protein. Scale bar: 20 μm (D) Fluorescence intensity of SSTR2 was measured using IMT i-Solution Industrial Image Analysis Software. (E) INS-1 cells were treated with 1 or 10 nmol/L of miR-16-5p inhibitor or negative control. miR-16-5p levels at 24 h after treatment were determined by qRT-PCR. (F) Western blotting of SSTR2, phospho-ERK (Thr202/Tyr204), ERK, and β-actin levels. The images shown here are cropped and the full-length original blots are shown in S8 Fig.

To identify the miRNAs involved in the early response of SSTR2 to octreotide treatment, INS-1 cells were treated with 1 μM octreotide for various incubation times. As shown in Fig 1A, SSTR2 expression was downregulated after 5 min of treatment with 1 μM octreotide. Immunocytochemistry revealed that membranous SSTR2 expression was reduced, and punctate perinuclear staining of SSTR2 was observed in the cytoplasm, suggesting that a 5-min incubation time is sufficient for identifying miRNAs associated with the early drug response of SSTR2 (Fig 1B). Thus, we analyzed the change in miRNA after 5 min octreotide treatment. miRNA array analysis showed that 21 miRNAs were downregulated and 9 were upregulated in INS-1 cells after 5-min treatment with octreotide compared with 0-min treatment (absolute fold-change >1.5; Fig 2A and 2B). Furthermore, KEGG pathway enrichment analysis revealed that the targets of these miRNAs were associated with cancer, mitogen-activated protein kinase (MAPK) signaling pathway, and endocrine and other factor-regulated calcium reabsorption (S1 Table). The expression of miR-16-5p, which showed the largest number of target genes among the 30 differentially expressed miRNAs, was verified by qPCR. Similar to the miRNA array results, miR-16-5p was upregulated after 5 min of octreotide treatment in INS-1 cells (Fig 2C). To confirm these results, we employed other neuroendocrine cells (GH3 cells). As shown in Fig 3D, miR-16-5p was upregulated in GH3 cells after 5 min incubation with octreotide. These results confirmed the effect of octreotide on the expression of miR-16-5p. Next, we investigated the function of miR-16-5p to SSTR2 expression after octreotide treatment.

### miR-16-5p regulates SSTR2 expression

To investigate the effect of miR-16-5p on SSTR2 expression, INS-1 cells were transfected with an miR-16-5p mimic to upregulate the expression of miR-16-5p; increased expression of miR-16-5p was confirmed by RT-qPCR (Fig 3A). Interestingly, total SSTR2 expression was significantly upregulated following the increase in miR-16-5p levels (Fig 3B). Moreover, the pERK/ERK ratio was upregulated in miR-16-5p–overexpressing INS-1 cells (Fig 3B); phosphorylation of ERK1/2 is predominantly associated with SSTR2 activation [12]. To examine the localization of increased SSTR2, immunocytochemistry was performed. Both membranous and cytoplasmic expression of SSTR2 was upregulated in miR-16-5p mimic-transfected cells (Fig 3C and 3D). We further employed a miR-16-5p inhibitor to downregulate miR-16-5p expression in INS-1 cells (Fig 3E) and found that INS-1 cells treated with the miR-16-5p inhibitor showed reduced miR-16-5p expression, resulting in downregulation of SSTR2 expression (Fig 3F). In addition, downregulated SSTR2 was observed in has-miR16-5p inhibitor-treated SSTR2-overexpressing HeLa cells by immunostaining (S2 Fig). Moreover, the pERK/ERK ratio was reduced after treatment with the inhibitor, suggesting reduced SSTR2 activation (Fig 3F). These results demonstrate that miR-16-5p is associated with the regulation of SSTR2 expression.

### miR-16-5p upregulation increases sensitivity to octreotide

**Fig 4:**
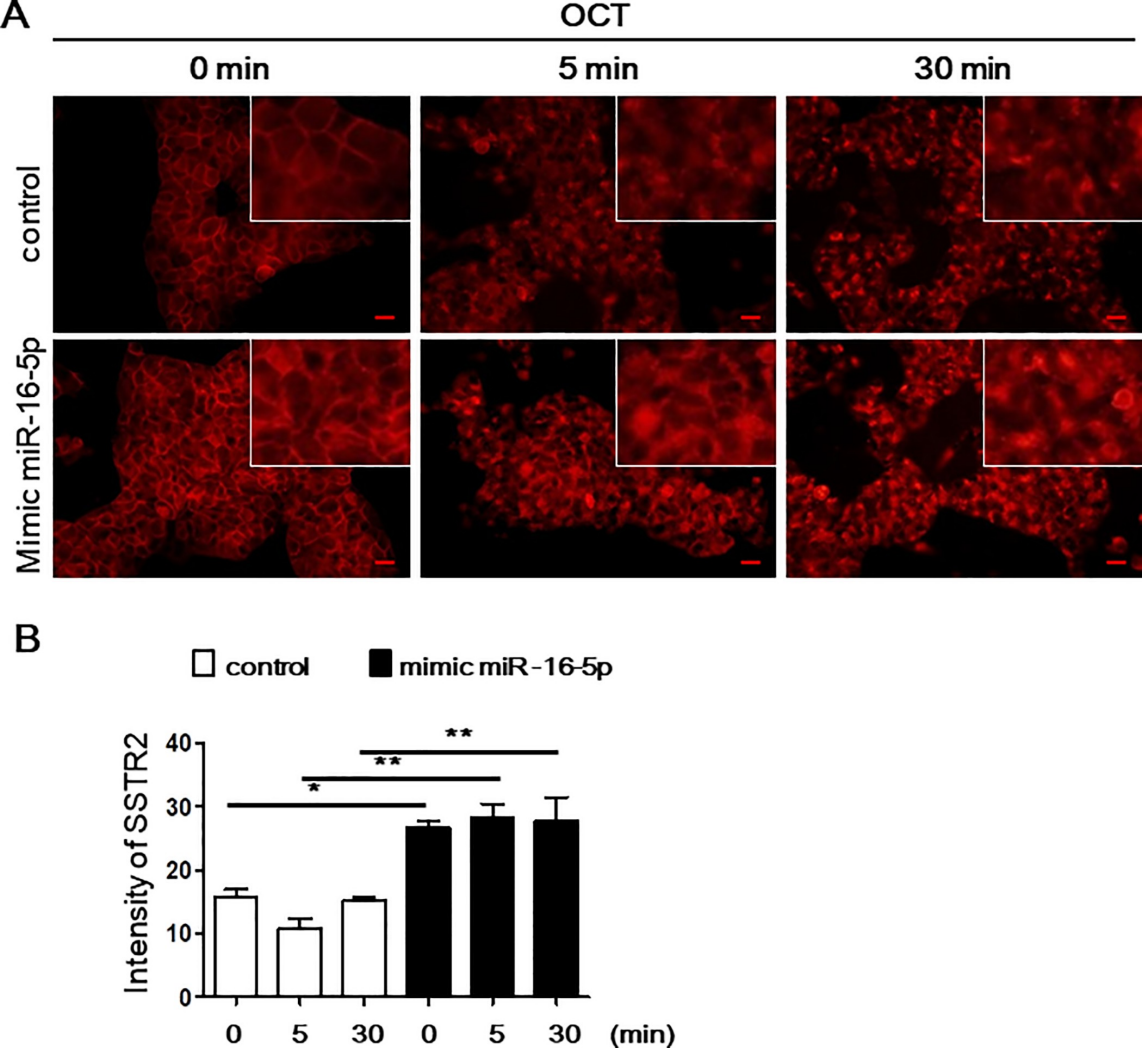
Upregulation of SSTR2 expression in INS-1 cells after octreotide treatment with mimic miR-16-5p. (A) INS-1 cells were transfected with 100 pmol of mimic miR-16-5p or control miRNA for 24 h and then treated with 1 μmol/L octreotide for the indicated durations. The cells were then fixed and processed for immunofluorescence staining of SSTR2 protein. Scale bar: 20 μm. (B) Fluorescence intensity of SSTR2 was measured with IMT i-Solution Industrial Image Analysis Software.

**Fig 5:**
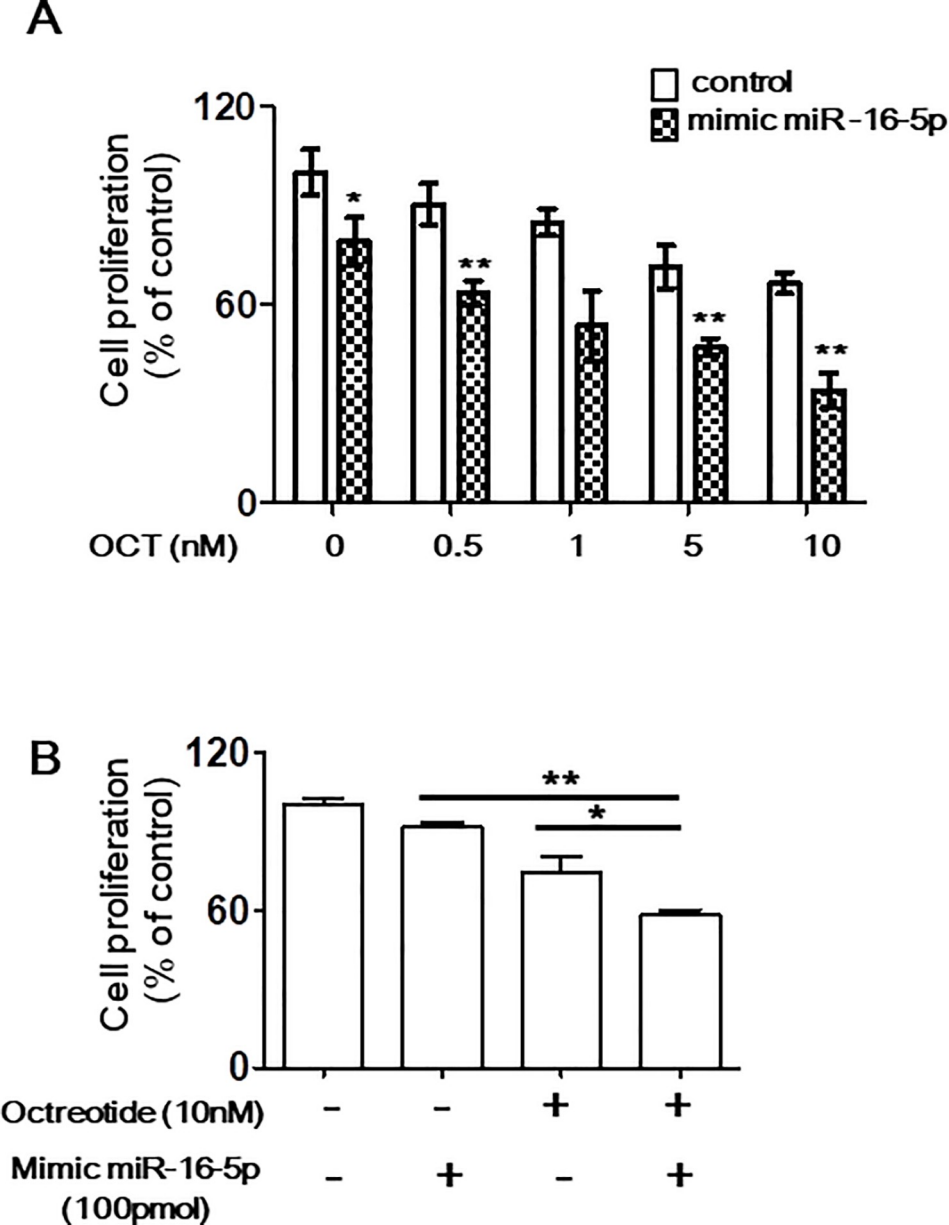
Sensitivity of INS-1 cells to octreotide after treatment with mimic miR-16-5p. (A) INS-1 cells were transfected with 2.5 pmol of mimic miR-16-5p or control miRNA for 24 h and treated with the indicated concentration of octreotide for 24 h. Cell proliferation was measured in a CCK-8 assay. (B) Spheroids from miR-16-5p mimic- or control-transfected INS-1 cells were treated with octreotide for 48 h. The proliferation of six spheroids were measured by CellTiter-Glo® 3D Cell Viability Assay. Values represent the mean of three independent experiments ± SD; *p < 0.05, **p < 0.01.

Because SSTR2 expression was found to be regulated by miR-16-5p, we assessed the effect of miR-16-5p on SSTR2 expression after octreotide treatment. INS-1 cells transfected with a miR-16-5p mimic or control miRNA were treated with octreotide for a short incubation time. Compared with control miRNA-transfected INS-1 cells, INS-1 cells exhibiting upregulation of miR-16-5p showed higher SSTR2 levels before and after octreotide treatment (Fig 4A and 4B). We next evaluated whether the increased SSTR2 levels induced by the miR-16-5p mimic affected the anti-cancer properties of octreotide. INS-1 cells transfected with the miR-16-5p mimic showed reduced cell proliferation after octreotide treatment (Fig 5A). To confirm the synergistic anti-cancer effect of combined treatment, annexin V-PI staining was performed. Annexin V-PI staining demonstrated that combined treatment with the mimic miR-16-5p and octreotide increased early and late apoptosis (S4 Fig). Spheroids from 3-dimensional (3D) culture systems have been used as models to evaluate drug sensitivity [13, 14]. Thus, we used spheroids to examine the role of miR-16-5p in octreotide sensitivity. The proliferation of miR-16-5p mimic-transfected INS-1 cells was reduced compared to that of spheroids from control miRNA-transfected INS-1 cells after 48 h octreotide treatment, demonstrating an increased sensitivity to octreotide (Fig 5B). In parallel, immunostaining for SSTR2 was performed. Similar to 2D culture, SSTR2 expression was upregulated and maintained in spheroids from miR-16-5p mimic-transfected INS-1 cells (S5 Fig). These findings suggest a role for miR-16-5p expression in mediating the antiproliferative action of octreotide.

## Discussion

The absence or low expression of SSTRs in NETs has been identified as the main cause of NET resistance to the current generation of SSAs. Therefore, to enhance NET cell sensitivity to octreotide, we used miRNA profiling and identified the miRNA associated with the initial response to octreotide treatment. Notably, our results showed that modulation of miR-16-5p levels affects SSTR2 expression and cell sensitivity to octreotide, suggesting that it can be used in combination with SSAs to improve their therapeutic effect via regulation of SSTR2 expression.

Few studies have determined the expression patterns of SSA-related miRNAs. Recently, Døssing et al. identified distinct dysregulation of miRNAs, including miR-7 and miR-148a, in the human neuroendocrine tumor cell line NCI-H727 after treatment with SSA [15]. Another study revealed that circulating miR-200a is involved in metastasis during SSA-treated small intestine NET progression [16]. However, little is known about the dysregulation of miRNAs during the early response to SSA. Thus, our study focused on miRNA expression patterns following treatment of cells with SSA under a shorter incubation period than that used in the two previous studies cited above. Only a few dysregulated miRNAs overlapped between our study and previous studies. Although the incubation time with octreotide was short, upregulation of miRNAs associated with the anticancer effect was found by KEGG analysis in our study. Moreover, our results showed that the predicted target genes of the 30 differentially expressed miRNAs were enriched in nine pathways, of which “microRNAs in cancer” and “MAPK signaling pathway” were the most prominent. Inhibition of cell proliferation by SSA through SSTRs involves several signal transduction pathways including control of the ERK/MAPK pathway [17]. Induction of ERK/MAPK pathway activation by the miR-16-5p-induced upregulation of SSTR2 was also observed in this study, suggesting that regulation of SSTR2 by miR-16-5p affects downstream signaling. Thus, we identified a miRNA involved in both early regulation of SSTR2 expression and the anticancer effect of octreotide.

Internalization of SSTR2 and intracellular trafficking are involved in SSTR desensitization. Recently, Gatto et al. suggested that low expression of ARRB1/β-arrestin 1 in pituitary adenoma is associated with reduced SSTR2 desensitization, leading to an improved response to SSA [18]. In our study, experiments focused on SSTR2 internalization and intracellular trafficking have not been performed. However, according to immunostaining, miR-16-5p overexpression may not only induce an increase in basal SSTR2 expression but also the internalization of SSTR2 under exposure to octreotide compared with that in the control. Indeed, miR-16-5p regulation in INS-1 cells also influenced the levels of Arrb1 mRNA, suggesting that miR-16-5p is involved in SSTR2 internalization and desensitization (S3 Fig).

SSTR2 regulation by chemical compounds has not been widely examined. Previous studies showed that histone deacetylase inhibitors and DNA methyltransferase inhibitors upregulate *Sstr2* in NET cells [19]. Our study revealed that upregulation of SSTR2 occurs partly via miR-16-5p in NET cells. Although we did not investigate the direct chemical compound targets of miR-16-5p, enrichment analysis suggested that some genes targeted by miR-16-5p are associated with the proteasome-ubiquitin system, including USP25. Thus, chemical compounds targeting the proteasome are potential candidates for modulating the expression of miR-16-5p. Therefore, further studies are needed to verify whether miR-16-5p directly binds to proteasome-related genes and investigate the effect of proteasome-related chemicals on miR-16-5p.

Several preclinical studies have shown that proteasome inhibitors impair proliferation and induce apoptosis in other neuroendocrine tumors such as pituitary tumors [20–22]. Thus, our results may be easy to apply in clinical settings to inhibit the reduction in SSTR2 expression by SSA. Moreover, SSTRs have been exploited as targets for NET diagnosis [23, 24]. Positron emission tomography imaging targeting SSTRs, such as imaging using ^68^Ga-labeled SSAs (DOTATOC, DOTATATE, and DOTANOC), was recently reported as a useful imaging modality for diagnosing NET and selecting patients for SSA treatment [25]. Thus, upregulation of SSTR2 by miR-16-5p may be effective in patients with low SSTR2 levels by enhancing positron emission tomography imaging-based diagnosis.

Collectively, our study highlights a novel role for miR-16-5p in regulating the expression of SSTR2 in NET cells. We also demonstrated that upregulation of mir16-5p enhances the sensitivity of octreotide in INS-1 cells, suggesting the potential of using octreotide with miR-16-5p as a novel combination therapy for NET treatment. Moreover, as desensitization and downregulation of SSTR2 after SSA treatment is an important clinical issue, the ability to modulate SSTR expression via miR-16-5p in patients with NET is clinically relevant, as it would improve the therapeutic response or detection sensitivity.

## Supporting information

S1 Fig Expression of SSTR2 in INS1 and GH3 cells. (A) INS1 and GH3 cells were plated on the coverslip. After 24 h, the cells were then fixed and processed for immunofluorescence staining of SSTR2 protein and counterstaining with DAPI.
(DOCX)

S2 Fig Expression of SSTR2 after treatment with has-miR-16-5p inhibitor in hSSTR2-transfected HeLa cells. HeLa cells were transfected with 1 μg of hSSTR2 or control plasmid for 24 h and then treated with the indicated concentration of has-miR16-5p inhibitor. (A) Cells were fixed and processed for immunofluorescence staining of SSTR2 protein. Scale bar: 50 μm. (B) Expression levels of has-miR-16-5p were determined after 24 h treatment by qRT-PCR. Data represent the mean of three independent experiments ± SD.
(DOCX)

S3 Fig Expression of *ARRB1* after modulation of miR-16-5p in INS1 cells. (A) Ins1 cells were transfected with mimic miR-16-5p or control. ARRB1 expression levels were determined after 24 h treatment by qRT-PCR. (B) (A) Ins1 cells were treated with miR16-5p inhibitor or control. ARRB1 expression levels were determined after 24 h treatment by qRT-PCR. Data represent the mean of three independent experiments ± SD.
(DOCX)

S4 Fig Analysis of apoptosis by Annexin V-APC/propidium iodide (PI) double staining of INS1 cells after treatment under the indicated conditions. Two-color flow cytometry dot plots show the percentages of living cells as negative for both annexin V and PI; early-stage apoptotic cells were Annexin V-positive and PI-negative, and late-stage apoptotic/necrotic cells were double-positive cells.
(DOCX)

S5 Fig INS-1 cells were transfected with 100 pmol of mimic miR-16-5p or control miRNA for 24 h and treated with 10 nmol/L octreotide for 24 h. Spheroids were then fixed with 4% PFA overnight at 4°C. Immunofluorescence staining of SSTR2 protein was performed. Scale bar: 100 μm.
(DOCX)

S6 Fig Full-length original blots of Fig 1A. We used ChemiDoc XRS (Biorad), which enables direct digital visualization of chemiluminescent western blots for the image of signals accumulated in the chemiluminescence reaction.
(DOCX)

S7 Fig Full-length original blots of Fig 3B. We used ChemiDoc XRS (Biorad), which enables direct digital visualization of chemiluminescent western blots for the image of signals accumulated in the chemiluminescence reaction.
(DOCX)

S8 Fig Full-length original blots of Fig 3F (Red box). We used ChemiDoc XRS (Biorad), which enables direct digital visualization of chemiluminescent western blots for the image of signals accumulated in the chemiluminescence reaction.
(DOCX)

S1 Table KEGG pathway enrichment analysis of miRNA targets in INS-1 cells after 5-min treatment with octreotide vs. 0-min treatment. (DOCX)
