# Supplementary material for: Elevated miR-16-5p induces somatostatin receptor 2 expression in neuroendocrine tumor cells
Source: PLoS One. 2020 Oct 12;15(10):e0240107. doi: 10.1371/journal.pone.0240107 (PMC7549806; doi:10.1371/journal.pone.0240107)
Supplement: S1 Fig — (A) INS1 and GH3 cells were plated on the coverslip. After 24 h, the cells were then fixed and processed for immunofluorescence staining of SSTR2 protein and counterstaining with DAPI. (DOCX) [file pone.0240107.s001.docx]

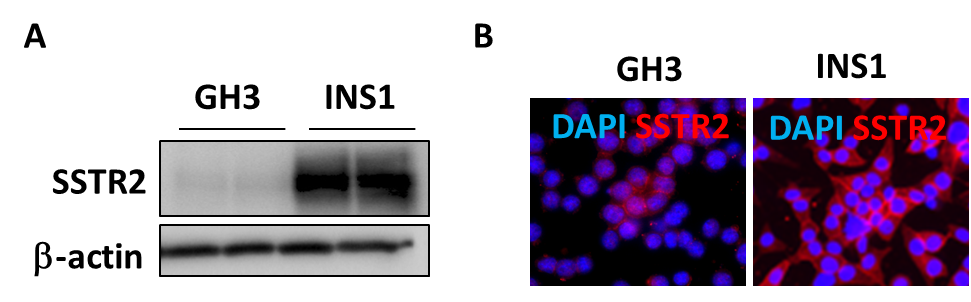


**Sup Fig 1. Expression of SSTR2 in INS1 and GH3 cells.** (A) INS1 and GH3 cells were plated on the coverslip. After 24 h, the cells were then fixed and processed for immunofluorescence staining of SSTR2 protein and counterstaining with DAPI.
