# Supplementary material for: Elevated miR-16-5p induces somatostatin receptor 2 expression in neuroendocrine tumor cells
Source: PLoS One. 2020 Oct 12;15(10):e0240107. doi: 10.1371/journal.pone.0240107 (PMC7549806; doi:10.1371/journal.pone.0240107)
Supplement: S2 Fig — HeLa cells were transfected with 1 μg of hSSTR2 or control plasmid for 24 h and then treated with the indicated concentration of has-miR16-5p inhibitor. (A) Cells were fixed and processed for immunofluorescence staining of SSTR2 protein. Scale bar: 50 μm. (B) Expression levels of has-miR-16-5p were determined after 24 h treatment by qRT-PCR. Data represent the mean of three independent experiments ± SD. (DOCX) [file pone.0240107.s002.docx]

**A**

**
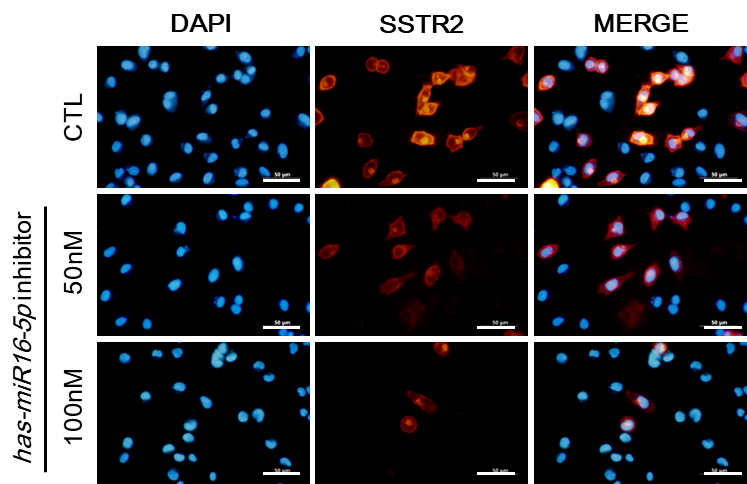
**


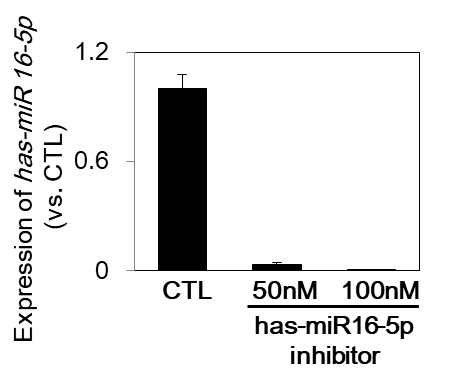
**B**

**Sup Fig 2.** **Expression of SSTR2 after treatment with has-miR-16-5p inhibitor in hSSTR2-transfected HeLa cells.** HeLa cells were transfected with 1 μg of hSSTR2 or control plasmid for 24 h and then treated with the indicated concentration of has-miR16-5p inhibitor. (A) Cells were fixed and processed for immunofluorescence staining of SSTR2 protein. Scale bar: 50 μm. (B) Expression levels of has-miR-16-5p were determined after 24 h treatment by qRT-PCR. Data represent the mean of three independent experiments ± SD.
