# Supplementary material for: Elevated miR-16-5p induces somatostatin receptor 2 expression in neuroendocrine tumor cells
Source: PLoS One. 2020 Oct 12;15(10):e0240107. doi: 10.1371/journal.pone.0240107 (PMC7549806; doi:10.1371/journal.pone.0240107)
Supplement: S3 Fig — (A) Ins1 cells were transfected with mimic miR-16-5p or control. ARRB1 expression levels were determined after 24 h treatment by qRT-PCR. (B) (A) Ins1 cells were treated with miR16-5p inhibitor or control. ARRB1 expression levels were determined after 24 h treatment by qRT-PCR. Data represent the mean of three independent experiments ± SD. (DOCX) [file pone.0240107.s003.docx]

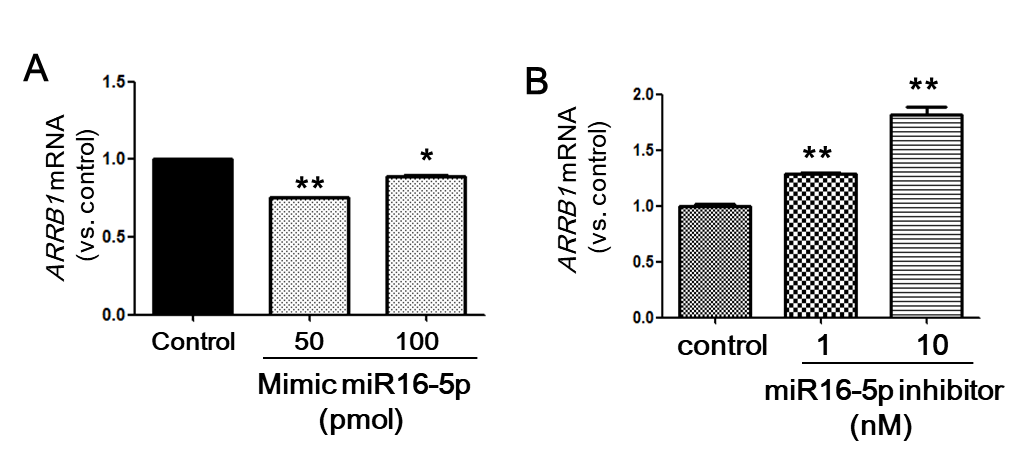


**Sup Fig 3.** **Expression of *ARRB1* after modulation of miR-16-5p in INS1 cells.** (A) Ins1 cells were transfected with mimic miR-16-5p or control. ARRB1 expression levels were determined after 24 h treatment by qRT-PCR. (B) (A) Ins1 cells were treated with miR16-5p inhibitor or control. ARRB1 expression levels were determined after 24 h treatment by qRT-PCR. Data represent the mean of three independent experiments ± SD.
