# Supplementary material for: Elevated miR-16-5p induces somatostatin receptor 2 expression in neuroendocrine tumor cells
Source: PLoS One. 2020 Oct 12;15(10):e0240107. doi: 10.1371/journal.pone.0240107 (PMC7549806; doi:10.1371/journal.pone.0240107)
Supplement: S4 Fig — Two-color flow cytometry dot plots show the percentages of living cells as negative for both annexin V and PI; early-stage apoptotic cells were Annexin V-positive and PI-negative, and late-stage apoptotic/necrotic cells were double-positive cells. (DOCX) [file pone.0240107.s004.docx]

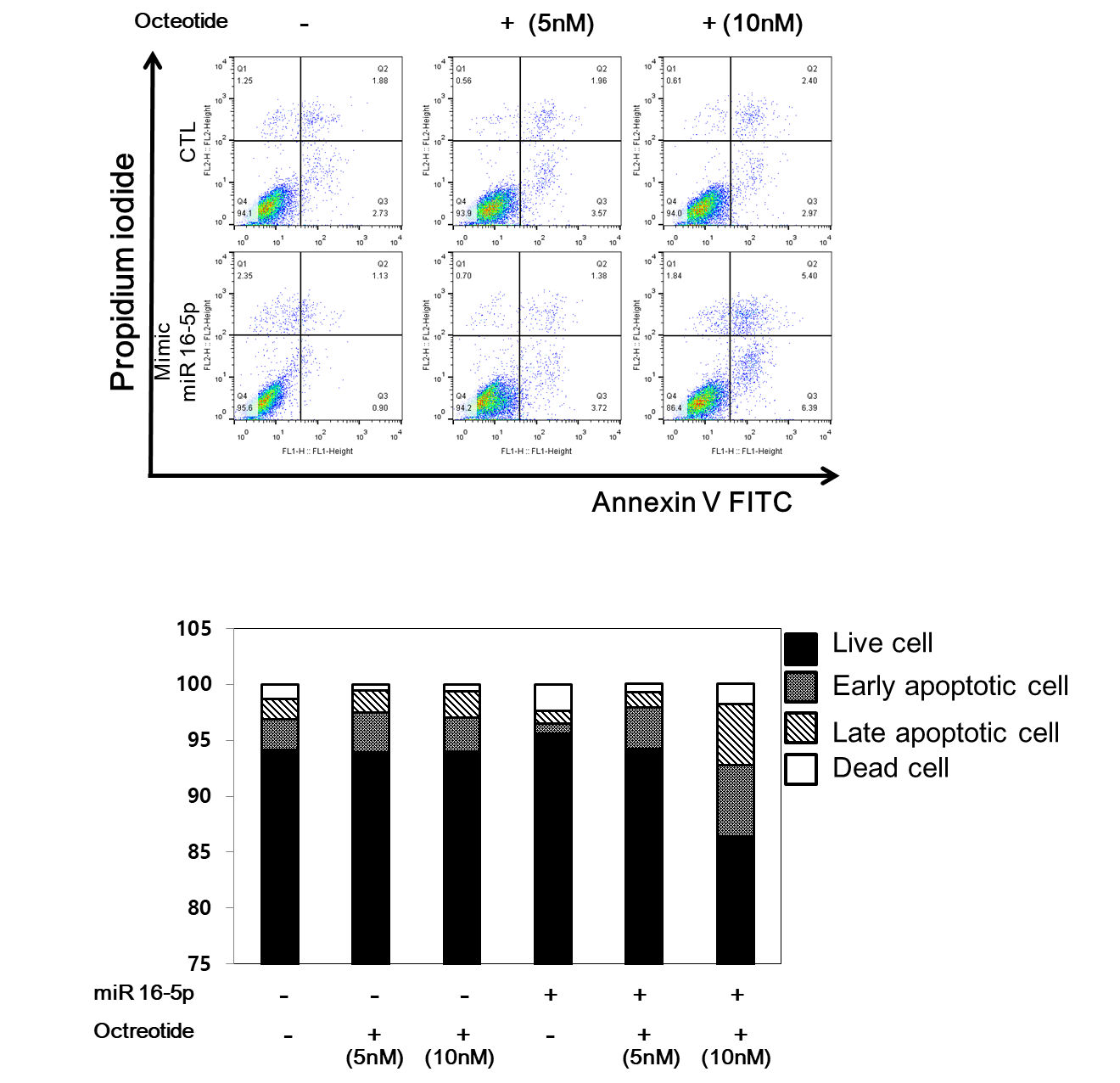


**Sup Fig 4. Analysis of apoptosis by Annexin V-APC/propidium iodide (PI) double staining of INS1 cells after treatment under the indicated conditions.** Two-color flow cytometry dot plots show the percentages of living cells as negative for both annexin V and PI; early-stage apoptotic cells were Annexin V-positive and PI-negative, and late-stage apoptotic/necrotic cells were double-positive cells.
