# Supplementary material for: Elevated miR-16-5p induces somatostatin receptor 2 expression in neuroendocrine tumor cells
Source: PLoS One. 2020 Oct 12;15(10):e0240107. doi: 10.1371/journal.pone.0240107 (PMC7549806; doi:10.1371/journal.pone.0240107)
Supplement: S5 Fig — Spheroids were then fixed with 4% PFA overnight at 4°C. Immunofluorescence staining of SSTR2 protein was performed. Scale bar: 100 μm. (DOCX) [file pone.0240107.s005.docx]

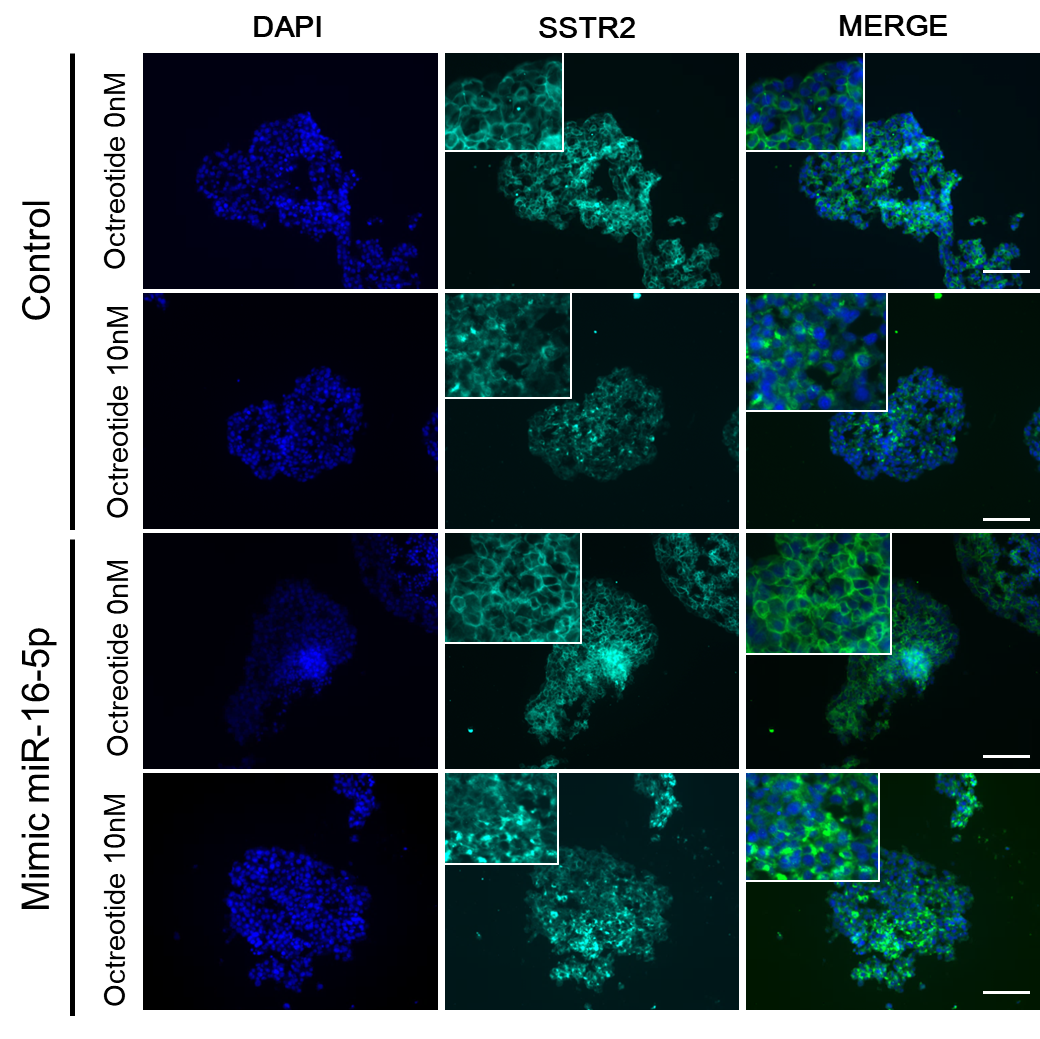


**Sup Fig 5.** INS-1 cells were transfected with 100 pmol of mimic miR-16-5p or control miRNA for 24 h and treated with 10 nmol/L octreotide for 24 h. Spheroids were then fixed with 4% PFA overnight at 4°C. Immunofluorescence staining of SSTR2 protein was performed. Scale bar: 100 μm.
