# Supplementary material for: Elevated miR-16-5p induces somatostatin receptor 2 expression in neuroendocrine tumor cells
Source: PLoS One. 2020 Oct 12;15(10):e0240107. doi: 10.1371/journal.pone.0240107 (PMC7549806; doi:10.1371/journal.pone.0240107)
Supplement: S7 Fig — We used ChemiDoc XRS (Biorad), which enables direct digital visualization of chemiluminescent western blots for the image of signals accumulated in the chemiluminescence reaction. (DOCX) [file pone.0240107.s007.docx]

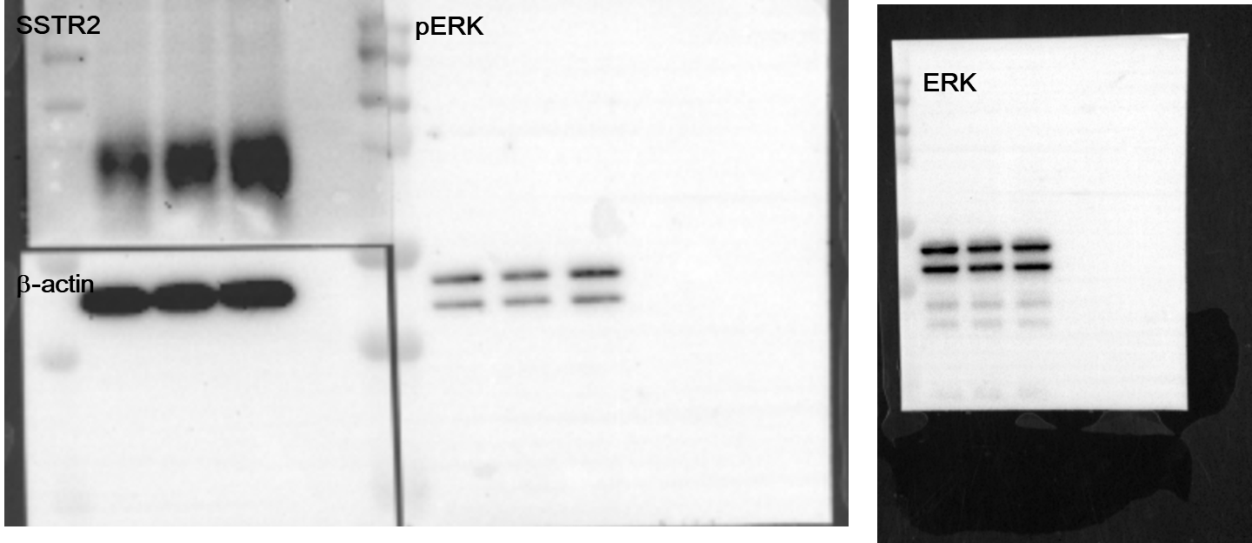


**Sup Fig 7.** Full-length original blots of Fig 3B. We used ChemiDoc XRS (Biorad), which enables direct digital visualization of chemiluminescent western blots for the image of signals accumulated in the chemiluminescence reaction.
