# Supplementary material for: Elevated miR-16-5p induces somatostatin receptor 2 expression in neuroendocrine tumor cells
Source: PLoS One. 2020 Oct 12;15(10):e0240107. doi: 10.1371/journal.pone.0240107 (PMC7549806; doi:10.1371/journal.pone.0240107)
Supplement: S8 Fig — We used ChemiDoc XRS (Biorad), which enables direct digital visualization of chemiluminescent western blots for the image of signals accumulated in the chemiluminescence reaction. (DOCX) [file pone.0240107.s008.docx]

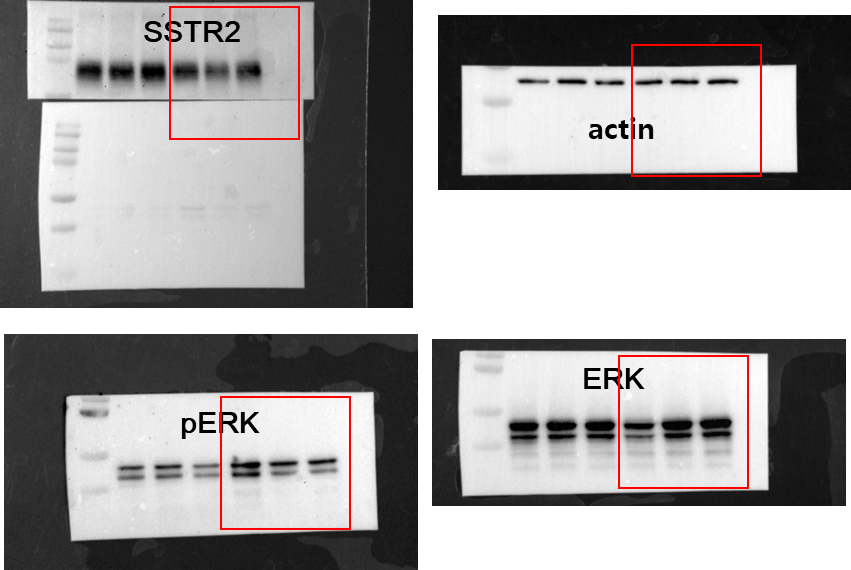


**Sup Fig 8.** Full-length original blots of Fig 3F (Red box). We used ChemiDoc XRS (Biorad), which enables direct digital visualization of chemiluminescent western blots for the image of signals accumulated in the chemiluminescence reaction.
