# Supplementary material for: Elevated miR-16-5p induces somatostatin receptor 2 expression in neuroendocrine tumor cells
Source: PLoS One. 2020 Oct 12;15(10):e0240107. doi: 10.1371/journal.pone.0240107 (PMC7549806; doi:10.1371/journal.pone.0240107)
Supplement: S1 Table — (DOCX) [file pone.0240107.s009.docx]

| **KEGG pathway** | **p-value** | **#genes** | **#miRNAs** |
| --- | --- | --- | --- |
| MicroRNAs in cancer | 1.28E-06 | 46 | 19 |
| MAPK signaling pathway | 3.38E-05 | 69 | 23 |
| FoxO signaling pathway | 0.000114 | 39 | 18 |
| Endocrine and other factor-regulated calcium reabsorption | 0.000366 | 12 | 9 |
| Vasopressin-regulated water reabsorption | 0.000413 | 15 | 10 |
| Gap junction | 0.000413 | 25 | 11 |
| Proteoglycans in cancer | 0.000498 | 50 | 20 |
| Transcriptional misregulation in cancer | 0.000795 | 44 | 19 |
| Wnt signaling pathway | 0.001226 | 39 | 17 |
| Pathways in cancer | 0.001711 | 83 | 23 |
| Mucin type O-Glycan biosynthesis | 0.004161 | 6 | 6 |
| Sphingolipid metabolism | 0.004161 | 15 | 11 |

**Sup table 1.** KEGG pathway enrichment analysis of miRNA targets in INS-1 cells after 5-min treatment with octreotide vs. 0-min treatment.
